# Association between the atherogenic index of plasma, body mass index, and cardiovascular diseases in Chinese middle and old-aged adults: a mediation analysis

**DOI:** 10.3389/fcvm.2025.1597749

**Published:** 2025-10-29

**Authors:** Yirui Xu, Mengping Liu, Wenbo Yang, Wenjiao Zhang, Heze Fan, Yu Xu, Juan Zhou, Zuyi Yuan

**Affiliations:** ^1^ Department of Cardiovascular Medicine, The First Affiliated Hospital of Xi’an Jiaotong University, Xi’an, Shaanxi, China; ^2^ Key Laboratory of Molecular Cardiology of Shaanxi Province, Xi'an Jiaotong University, Xi’an, Shaanxi, China

**Keywords:** atherogenic index of plasma, body mass index, cardiovascular diseases, CHARLS, mediation analysis

## Abstract

**Background:**

Evidence has emerged that suggests the atherogenic index of plasma (AIP), as determined by blood lipid levels, is a valuable predictor of the onset of cardiovascular diseases. The correlation between AIP and cardiovascular diseases may be facilitated by body mass index (BMI), as atherosclerosis is prevalent in overweight and obese populations. In order to assess the combined predictive value of the AIP and BMI in predicting outcomes associated with cardiovascular diseases, this study sought to determine if BMI mediates the relationship between the AIP and cardiovascular disease risk.

**Methods:**

Participants in this study were those who were without cardiovascular diseases at baseline and were members of the China Health and Retirement Longitudinal Study (CHARLS). We performed adjusted regression analysis and mediation. AIP was calculated using the logarithm of the ratio of triglycerides (TG) to high-density lipoprotein cholesterol (HDL-C). The estimated body weight and height were used to calculate the BMI.

**Results:**

This study included 5468 participants. Cardiovascular diseases were highly linked with both BMI and AIP. In contrast to those with a lower AIP (median level) and BMI <24, those with an elevated AIP and increased BMI presented a higher risk for developing cardiovascular conditions (HR: 1.789; 95% CI: 1.491–2.147). Additionally, about 30.57% of the connection between AIP and cardiovascular diseases was strongly mediated by BMI.

**Conclusions:**

Among Chinese adults in their middle and advanced years, the AIP and BMI jointly have been associated to a higher risk of cardiovascular diseases, and BMI could serve as a mediator in this connection.

## Introduction

Cardiovascular diseases remain one of the highest-incidence types of diseases, and their mortality and disability rates place enormous burdens on human health and the economy (1). As a group of atherosclerotic-related disorders, Cardiovascular diseases are driven by multiple modifiable risk factors, among which dyslipidemia and obesity stand out for their high prevalence and strong causal links to disease progression. Dyslipidemia, characterized by abnormal lipid metabolism, is widely recognized as a core driver of atherosclerotic plaque formation—an early and critical step in cardiovascular diseases pathogenesis (2). The atherogenic index of plasma (AIP), proposed in 2001, has emerged as a more comprehensive marker of atherogenic risk than single lipid parameters. Unlike isolated lipid measurements, AIP integrates TG and HDL-C to reflect the balance between pro- and anti-atherogenic lipids, and accumulating evidence confirms its association with key atherosclerotic phenotypes: higher AIP levels correlate with increased carotid intima-media thickness, accelerated plaque progression, and elevated incidence of carotid atherosclerosis (3–6).

Obesity, often assessed via body mass index (BMI), is another major cardiovascular diseases risk factor, primarily through its role in triggering insulin resistance, chronic low-grade inflammation, and metabolic dysregulation (7, 8). As the most widely used indicator of overweight and obesity, BMI correlates strongly with total body fat mass (9, 10) and is explicitly incorporated into metabolic syndrome diagnostic criteria by the World Health Organization (WHO) and Chinese Diabetes Society (CDS) criteria for metabolic syndrome (11).

Notably, both AIP and BMI have been independently linked to cardiovascular diseases risk in prior studies. However, existing research on their interaction remains limited and focused on specific outcomes: for instance, a national prospective cohort study in middle-aged and older Chinese adults explored the association between AIP and obesity-related stroke (12), confirming a synergistic role of these two factors in stroke risk. Nevertheless, critical gaps persist: few studies have systematically analyzed the mechanistic pathway connecting AIP to broad cardiovascular diseases outcomes, particularly whether BMI mediates the AIP and cardiovascular diseases association. Quantifying such a mediating effect could clarify how lipid-related atherogenic risk intersects with obesity-driven metabolic dysfunction to influence cardiovascular diseases, insights that may inform targeted interventions, such as addressing insulin resistance to mitigate both lipid abnormalities and obesity-related risk.

Against this background, the present study aimed to: (1) investigate the combined association of AIP and BMI with incident cardiovascular diseases in middle-aged and older Chinese adults; (2) test the hypothesis that BMI mediates the relationship between AIP and cardiovascular diseases. By addressing these objectives, we sought to refine cardiovascular diseases risk stratification and provide evidence for more precise prevention strategies.

## Materials and methods

### Study population

Information collected by the China Health and Retirement Longitudinal cohort (CHARLS), a longitudinal study of Chinese citizens aged 45 and over that is nationally representative, is secondary analyzed in this study. Detailed information about the study design of the CHARLS is provided in the Supplementary Methods.

**Figure 1:**
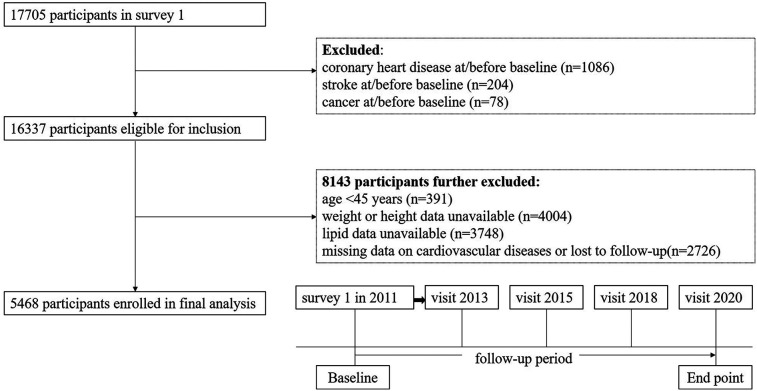
Flowchart and follow-up setting of this current study.

The 2011 baseline survey had 17,705 respondents, who were then contacted for the four follow-up surveys. We excluded 12,237 participants for the following reasons: (1) history of coronary heart disease (*n* = 1,086); (2) history of stroke (*n* = 204); (3) personal history of cancer (*n* = 78); (4) under the age of 45 or those with missing age-related data (*n* = 391); (5) missing data on height or weight (*n* = 4,004); (6) missing TG or HDL-C levels (*n* = 3,748); and (7) missing data on cardiovascular diseases or “lost to follow-up” (*n* = 2,726). Ultimately, a cohort of 5,468 participants were included for longitudinal analysis. The details are shown in Figure 1.

### Exposure and outcome

The interviewer utilized a standardized questionnaire to capture demographic data, health status, and participants' functional capacity. The AIP, that is the negative logarithm of the TG to HDL-C ratio, BMI was calculated by qualified nurses by dividing weight in kilos by height in meters squared.

The primary endpoint of the trial was defined as the occurrence of cardiovascular diseases, specifically encompassing the incidence of heart disease or stroke during the follow-up period. (for further information, see Supplementary Methods) (13). Participants reporting heart disease or stroke during the follow-up period were considered to have cardiovascular diseases, which are validated and widely available.

### Covariates

We added potentially confounding factors at baseline in accordance with earlier research. Age, sex, and levels of education were among the demographic confounders. The health behavior covariates included smoking status and self-reported health conditions (hypertension, diabetes). Glycosylated hemoglobin (HbA1c), estimated glomerular filtration rate (eGFR), and uric acid were among the laboratory test results. The Supplementary Methods section has comprehensive details on the covariates.

### Statistical analysis

The fundamental characteristics were described using the percentages of classified data as well as the means and standard deviations (SDs) of the continuous data. Continuous variables were analyzed using the Kruskal–Wallis rank sum test, whereas categorical variables were examined using either the chi-square test or Fisher's exact test, as appropriate. To investigate the associations between the AIP, BMI, and the incidence of cardiovascular diseases, multiple linear regression analysis was employed to evaluate the relationships between BMI and AIP.

Regression coefficients (*β*) with 95% CIs were utilized to indicate the effect sizes. The hazard ratio (HR) with a 95% confidence interval (CI) was then computed using multivariable-adjusted Cox regression models, taking the time-to-event frame into account. No variables were taken into account when creating the rough model. The adjusted model included the following variables: age (continuous), sex (female, male), education level (primary school or lower, high school or higher), smoking status (never smoked, smoking), diabetes status (yes, no), hypertension status (yes, no), HbA1c level (continuous), uric acid level (continuous), and eGFR (continuous). Based on the median value, the AIP was split into two groups. BMI was divided into two groups: <24 kg/m^2^ and ≥24 kg/m^2^, with overweight and obesity being classified as BMI ≥ 24 kg/m^2^. Following that, the subjects were split into four groups based on the combined evaluation of their BMI and AIP. Dose-response relationships involving the AIP and BMI and cardiovascular risk were demonstrated using the limited cubic spline function, which featured four knots at the 20th, 40th, 60th, and 80th percentiles. The reference point was established as the median value of the variables in the associated population. After further correcting for hs-CRP (continuous) and defining hypertension as 130/80 mmHg, we conducted a number of sensitivity analyses. Additionally, propensity scores derived from the inverse probability of treatment weighting (IPTW) method were utilized to reassess the impact of BMI and AIP on cardiovascular risk. The participants were divided into different subgroups according to age, sex, education level, smoking and drinking histories, and subgroup analysis in order to evaluate the findings' robustness. The area under the receiver operating characteristic curve (ROC) and decision curve were used to assess the predictive usefulness of the AIP and BMI separately or in combination for cardiovascular diseases. The net reclassification index (NRI) was used to compare whether the AIP and BMI together were more predictive of cardiovascular diseases than the AIP and BMI alone.

To assess the direct and indirect relationships between AIP and cardiovascular diseases through elevated BMI, we performed a mediation study. The Supplementary Methods section has comprehensive details on the covariates.

Every statistical study was conducted using R software, version 4.4.1. The “mediation” package was used for the mediation analysis, whereas the “ipw” package was used to implement IPTW. A two-sided *P*-value of less than 0.05 was considered statistically significant.

## Results

### Population characteristics

The baseline clinical and demographic data of the participants, grouped by AIP and BMI are shown in Table 1, and those of the participants grouped by cardiovascular diseases are shown in Supplementary Table S1 and Table 1 shows the final analysis comprised a total of 5,468 participants from the 2011–2020 CHARLS project. There were 2,957 (54.1%) females and a mean (SD) age of 58.2 (8.6) years. 1,248 (22.8%) people got cardiovascular diseases throughout a maximum follow-up time of 9.0 years, including 1,074 (19.6%) instances of heart disease and 447 (8.2%) cases of stroke. There were 1,921 (35.3%) patients with hypertension and 839 (15.5%) with diabetes. Supplementary Table S1 shows that among those who developed cardiovascular diseases during the follow-up period, weight, BMI, and AIP significantly increased. According to Table 1, the group with higher AIP and BMI had higher rates of diabetes and hypertension. Furthermore, whereas HDL-C levels were lower in these groups, HbA1c, waist circumference, total cholesterol (TC), systolic blood pressure (SBP), fasting plasma glucose, diastolic blood pressure (DBP), TG, and uric acid levels were higher.

**Table 1:** Characteristics of 5,468 participants categorized by AIP and BMI levels.

Variables	Overall	AIP < median & BMI < 24	AIP < median & BMI ≥ 24	AIP ≥ median & BMI < 24	AIP ≥ median & BMI ≥ 24	*P*-Value
Participants, No	5,468	2,030	704	1,281	1,453	
Age, years, mean (SD)	58.2 (8.6)	59.3 (8.9)	56.3 (8.3)	59.1 (8.7)	56.7 (7.9)	<0.001
Sex, Female, *n* (%)	2,957 (54.1)	963 (47.5)	455 (64.6)	655 (51.2)	884 (60.8)	<0.001
Residence, *n* (%)						<0.001
Rural	4,728 (86.5)	1,828 (90.0)	585 (83.2)	1,135 (88.6)	1,180 (81.3)	
Urban	738 (13.5)	202 (10.0)	118 (16.8)	146 (11.4)	272 (18.7)	
Marriage, married, *n* (%)	4,940 (90.3)	1,797 (88.5)	646 (91.8)	1,134 (88.5)	1,363 (93.8)	<0.001
Educational level, *n* (%)						0.002
High school or higher	524 (9.6)	163 (8.0)	84 (11.9)	114 (8.9)	163 (11.2)	
Primary or lower	4,943 (90.4)	1,866 (92.0)	620 (88.1)	1,167 (91.1)	1,290 (88.8)	
Smoking status, *n* (%)						<0.001
Never	3,391 (62.1)	1,134 (55.9)	526 (74.7)	734 (57.4)	997 (68.6)	
Smoking	2,073 (37.9)	895 (44.1)	178 (25.3)	544 (42.6)	456 (31.4)	
Drinking status, *n* (%)						0.235
Never	3,751 (92.6)	1,304 (92.4)	502 (94.7)	891 (92.5)	1,054 (92.0)	
Drinking	300 (7.4)	108 (7.6)	28 (5.3)	72 (7.5)	92 (8.0)	
Hypertension, *n* (%)	1,921 (35.3)	512 (25.3)	286 (40.9)	418 (32.9)	705 (54.1)	<0.001
Diabetes, *n* (%)	839 (15.5)	179 (8.9)	82 (11.8)	226 (17.8)	352 (24.4)	<0.001
Height, cm, mean (SD)	157.9 (8.5)	157.9 (8.7)	156.8 (7.9)	158.1 (8.5)	158.3 (8.2)	0.001
Weight, kg, mean (SD)	58.6 (11.0)	52.2 (7.6)	65.5 (9.4)	53.9 (7.5)	68.2 (9.5)	<0.001
Waist, cm, mean (SD)	84.9 (9.8)	78.6 (6.8)	91.5 (7.4)	81.1 (6.9)	93.9 (7.6)	<0.001
SBP, mmHg, mean (SD)	128.7 (20.5)	124.5 (19.6)	131.2 (20.4)	128.0 (20.4)	134.0 (20.6)	<0.001
DBP, mmHg, mean (SD)	75.2 (11.9)	72.1 (11.2)	77.6 (11.8)	74.2 (11.6)	79.1 (11.7)	<0.001
Glucose, mg/dl, mean (SD)	109.2 (34.3)	102.8 (22.8)	104.6 (27.2)	111.9 (40.4)	118.0 (41.8)	<0.001
HbA1c, %, mean(SD)	5.2 (0.8)	5.2 (0.6)	5.2 (0.7)	5.2 (0.8)	5.4 (0.9)	<0.001
C-Reactive Protein, mg/dl, mean (SD)	2.4 (6.7)	2.3 (7.6)	2.5 (6.1)	2.4 (7.5)	2.5 (4.4)	0.765
Creatinine, mg/dl, mean (SD)	0.8 (0.2)	0.8 (0.2)	0.7 (0.2)	0.8 (0.2)	0.8 (0.2)	<0.001
Uric acid, mg/dl, mean (SD)	4.4 (1.2)	4.2 (1.1)	4.3 (1.2)	4.5 (1.3)	4.6 (1.2)	<0.001
eGFR, ml/min/1.73m^2^, mean (SD)	97.0 (13.4)	97.5 (12.6)	98.5 (13.1)	95.6 (14.2)	96.8 (13.9)	<0.001
Triglycerides, mg/dl, mean (SD)	132.5 (112.9)	74.5 (21.8)	78.2 (20.9)	180.6 (126.9)	197.5 (143.3)	<0.001
Total Cholesterol, mg/dl, mean (SD)	193.8 (38.7)	188.6 (34.9)	190.4 (35.3)	195.0 (40.7)	201.4 (42.0)	<0.001
HDL Cholesterol, mg/dl, mean (SD)	51.5 (15.2)	61.9 (14.1)	57.6 (12.0)	43.6 (10.4)	41.1 (9.4)	<0.001
LDL Cholesterol, mg/dl, mean (SD)	116.4 (34.7)	114.0 (30.8)	120.0 (31.2)	114.4 (37.6)	119.8 (38.2)	<0.001
AIP, mean (SD)	0.8(0.8)	0.2(0.4)	0.3(0.3)	1.3(0.6)	1.5(0.6)	<0.001
CVD, *n* (%)	1,248(22.8)	351(17.3)	179(25.4)	302(23.6)	416(28.6)	<0.001

Data are presented as mean (SD) or number (%), as appropriate.

SD, standard deviation; AIP, atherogenic index of plasma; BMI, body mass index; SBP, systolic blood pressure; DBP, diastolic blood pressure; HbA1c, glycosylated hemoglobin; eGFR, estimated glomerular filtration rate; LDL, low-density lipoprotein; HDL, high-density lipoprotein; CVD, cardiovascular diseases.

### Associations of AIP with BMI

The relationship between AIP and BMI levels is seen in Table 2. The participants with greater AIP had significantly higher BMI levels after controlling for covariates (AIP ≥ median: *β*: 0.227, 95% CI: 0.047–0.406). Supplementary Figure S1 shows that there is a notable increase in BMI in tandem with an increase in AIP.

**Table 2:** Association between AIP and BMI using multiple linear regression.

AIP	Unadjusted		Adjusted	
	*β* (95% CI)	*P* value	*β* (95% CI)	*P* value
AIP < median	0 (Reference)		0 (Reference)	
AIP ≥ median	0.296 (0.111–0.480)	<0.001	0.227 (0.047–0.406)	<0.001

The median AIP value is 0.72. Age, sex, education level, smoking status, hypertension, diabetes, HbA1c, uric acid, and eGFR were adjusted.

### Associations of the AIP, and BMI with cardiovascular diseases

**Figure 2:**
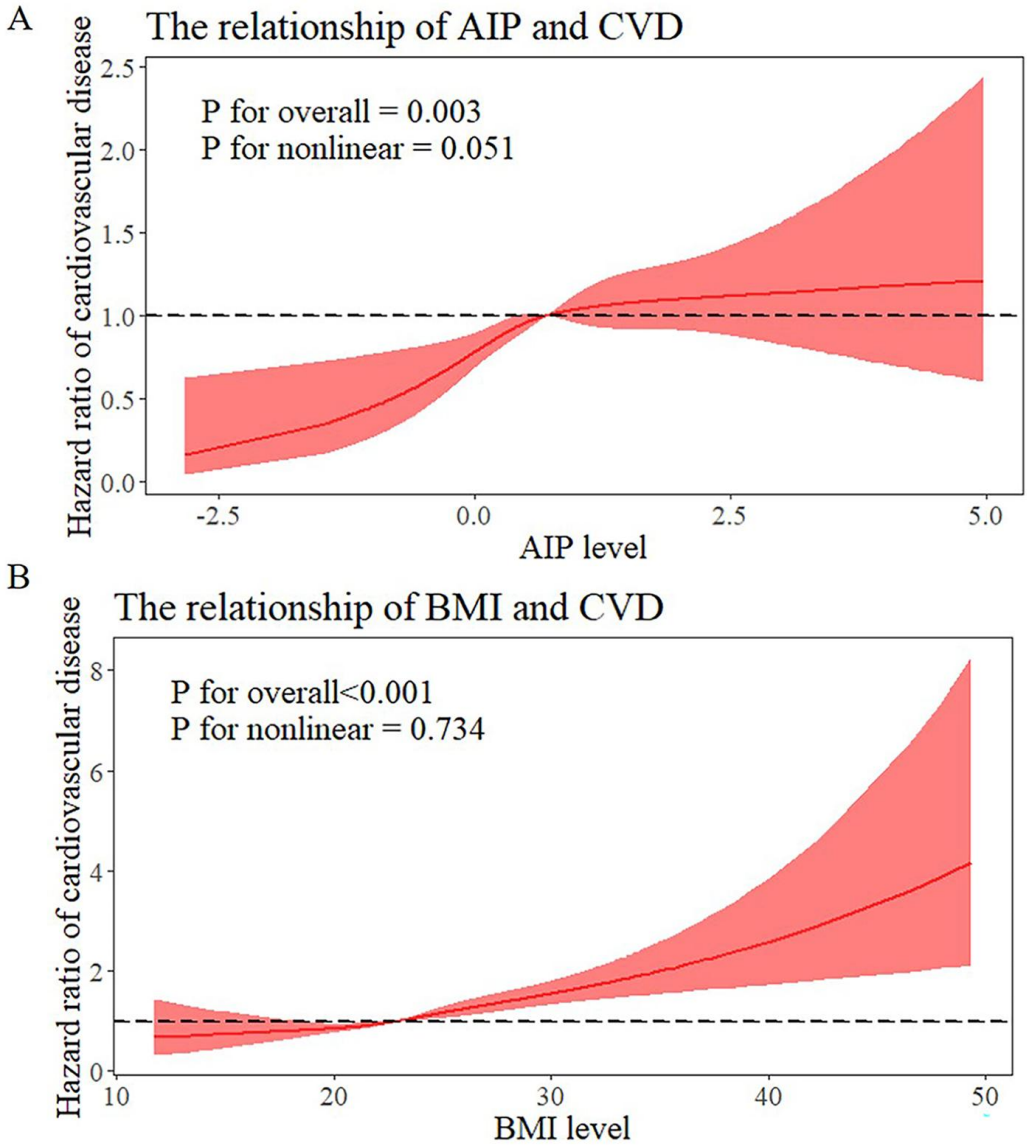
Dose-responsive relationship of the AIP and BMI levels with the risk of cardiovascular diseases. **(A)** Dose-responsive relationship of the AIP and the risk of cardiovascular diseases. **(B)** Dose-responsive relationship of the BMI and the risk of cardiovascular diseases.

**Figure 3:**
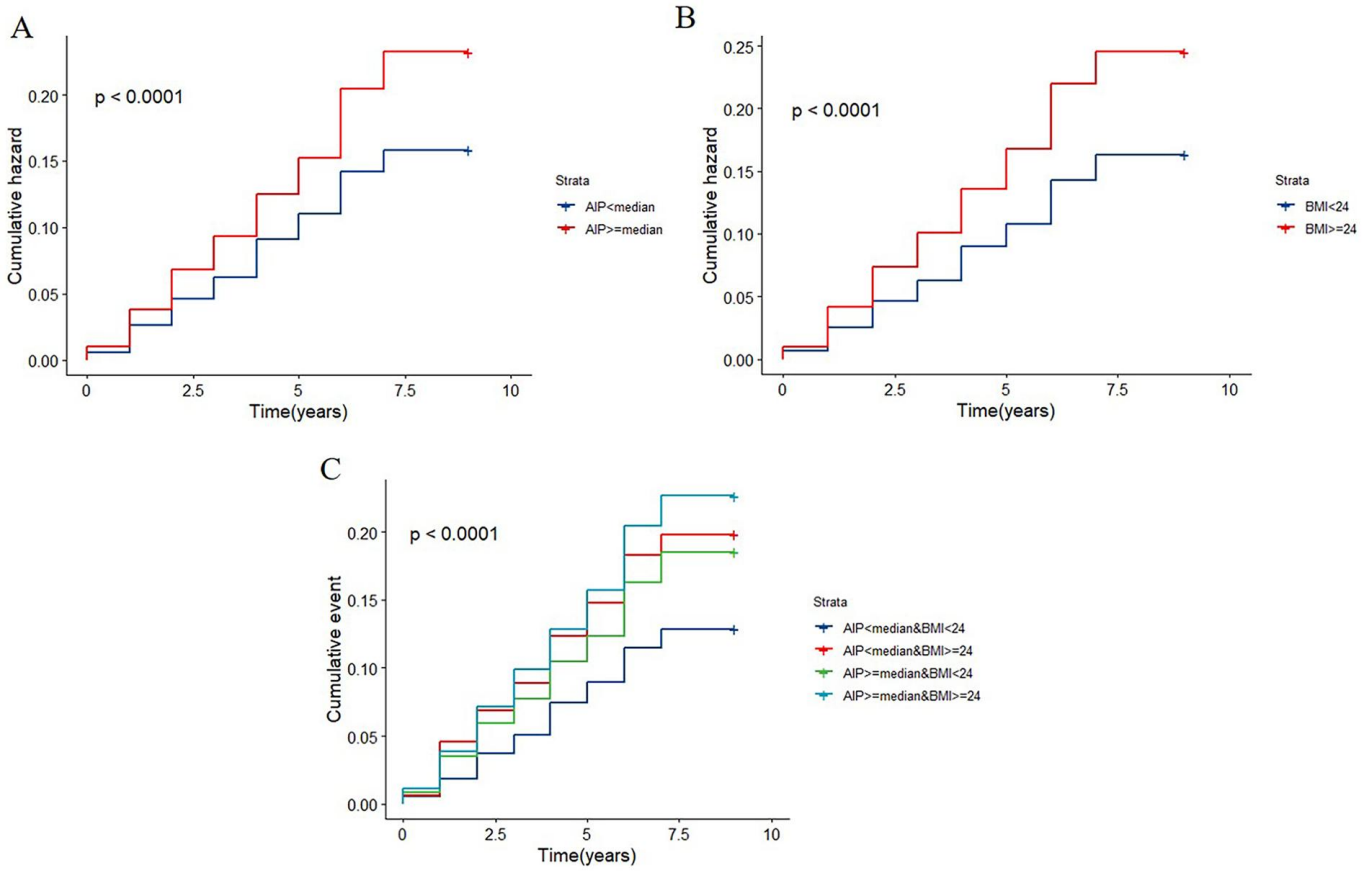
K–M plot of cardiovascular diseases by AIP and BMI levels. **(A)** The cumulative hazard of cardiovascular disease by AIP strata (AIP < median vs. AIP ≥ median). **(B)** The cumulative hazard by BMI strata (BMI < 24 vs. BMI ≥ 24). **(C)** The cumulative hazard by combined AIP and BMI strata.

The relationship between the AIP and incident cardiovascular disease risk is seen in Supplementary Table S2. After adjustment for confounders, the risk of cardiovascular diseases among individuals with AIP ≥ median was found to be 1.359 (95% CI: 1.184–1.562) times higher compared to those with AIP < median. The relationships between BMI and the risk of cardiovascular diseases are also shown in Supplementary Table S2. Participants who were overweight or obese had higher risks of cardiovascular diseases than those who were normal weight after controlling for covariates (HR: 1.483; 95%CI: 1.287–1.708). A significant dose-response relationship between the risk of cardiovascular diseases and the AIP and BMI as continuous variables was also found by multivariable-adjusted restricted cubic splines analysis. Additionally, when these variables were examined as continuous variables, there was a positive correlation between the AIP and BMI and cardiovascular diseases (Figures 2A,B). When analyzed as a categorical variable, the group with a higher index (AIP ≥ median) presented a significantly greater cardiovascular disease cumulative risk within the same period than did the group with a lower index (AIP < median), and these differences were highly statistically significant. The same phenomenon was also observed for BMI (Figures 3A,B).

### Joint analysis of AIP and BMI for cardiovascular diseases

When the baseline AIP and BMI were evaluated jointly, the cumulative incidence rates of cardiovascular diseases are displayed in Figure 3C. Those with greater AIP and BMI had the greatest rates of cardiovascular diseases. As presented in Table 3, participants with elevated AIP and higher BMI showed the greatest cardiovascular disease risk in the fully adjusted analysis, relative to individuals with AIP below the median and BMI less than 24 kg/m^2^. These individuals were followed by those with a lower AIP and increased BMI, and those with a higher AIP and BMI < 24 kg/m^2^. Supplementary Tables S4 and S5 demonstrate that regardless of whether stroke is included in the definition of cardiovascular diseases, the groups with elevated AIP and elevated BMI consistently exhibit the highest risk of endpoint events.

**Table 3:** Associations of AIP and BMI levels with risk of cardiovascular diseases.

Group	Unadjusted		Adjusted	
	HR (95% CI)	*P* value	HR (95% CI)	*P* value
AIP < median & BMI < 24	Ref		Ref	
AIP < median & BMI ≥ 24	1.618 (1.309–2.001)	<0.001	1.653 (1.327–2.058)	<0.001
AIP ≥ median & BMI < 24	1.488 (1.242–1.783)	<0.001	1.418 (1.180–1.705)	<0.001
AIP ≥ median & BMI ≥ 24	1.866 (1.578–2.207)	<0.001	1.789 (1.491–2.147)	<0.001

BMI was calculated by dividing an individual's weight in kilograms by the square of their height in meters; the unit of BMI: kg/m^2^; the median value of the AIP: 0.72. Age, sex, education level, smoking status, hypertension, diabetes, HbA1c, uric acid, and eGFR were adjusted; HR, hazard ratio; CI, confidence interval; BMI, body mass index; AIP, atherogenic index of plasma.

### Sensitivity and subgroup analyses

**Figure 4:**
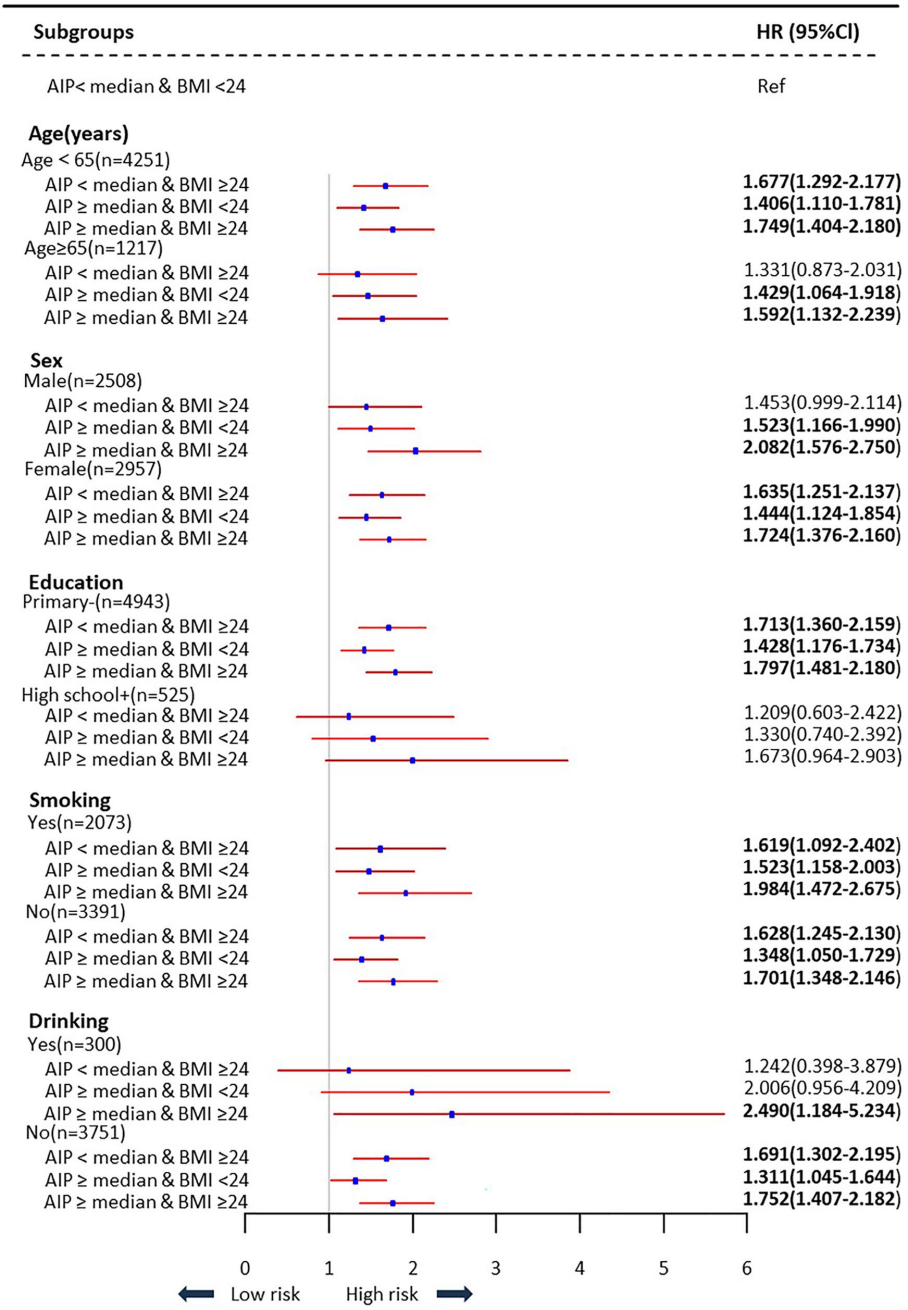
Subgroups analysis of association between AIP and BMI levels with the risk of cardiovascular diseases.

Supplementary Table S3 summarizes the findings, which held true across several sensitivity analyses. When the definitions of hypertension and hs-CRP were further modified, the highest HR values were virtually the same. The results were largely consistent, and the effect was amplified when propensity scores were combined with the IPTW approach (Supplementary Table S3). Supplementary Table S6 demonstrates that the use of different BMI cut-off values does not alter our core conclusion, which is that higher AIP and higher BMI are associated with increased cardiovascular diseases risk in Chinese adults. Subgroup analyses based on age, sex, education level, smoking status, and drinking status were used to look at the relationships between AIP and BMI and cardiovascular diseases across a variety of subgroups. The findings indicated that the groupings with the highest risk of cardiovascular diseases were those with greater BMI and elevated AIP out of all individuals examined. However, in the grouping with higher educational attainment, no statistically significant difference was found (Figure 4).

### Predictive value of the AIP and BMI for cardiovascular diseases

**Figure 5:**
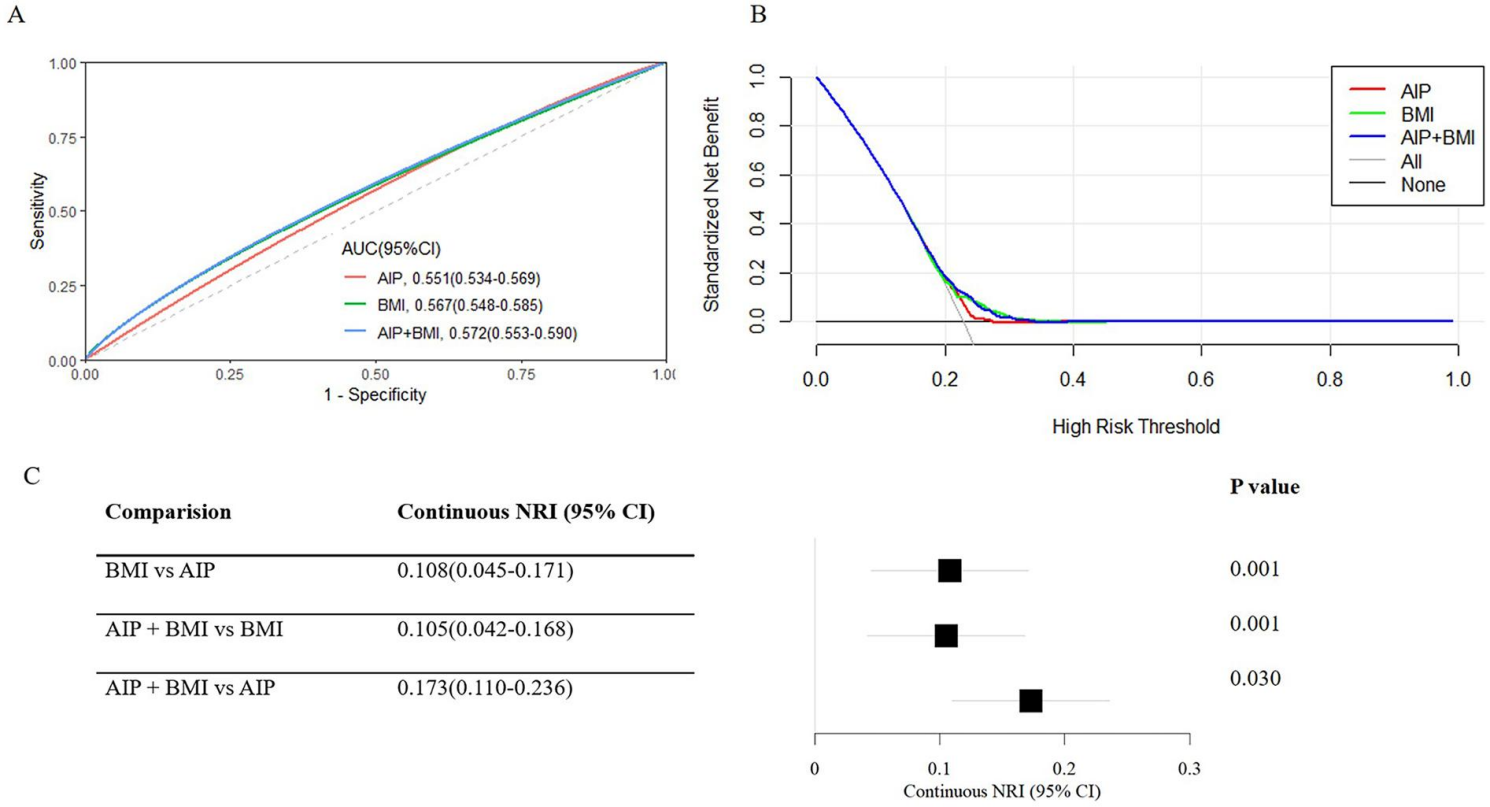
Predictive performance of the combined AIP and BMI for cardiovascular diseases. **(A)** The receiver operating characteristic (ROC) curve evaluates the discriminative capabilities by calculating the AUC. **(B)** Decision curve analysis to compare the clinical utility, the y-axis represents net benefits, calculated by subtracting the relative harm (false positives) from the benefits (true positives). The x-axis calculates the threshold probability. **(C)** NRI index for AIP combined with BMI. AIP, Atherogenic index of plasma; AUC, Area under curve; BMI, Body mass index; CI, Confidence interval; NRI, Net reclassification index.

We compared their performance to the enhanced prediction value of combining the AIP and BMI. The ROC curve showed that the combined metric's area under the curve (AUC) was 0.572 (95% CI: 0.553–0.590) (Figure 5A), and its clinical importance was confirmed by the decision curve (Figure 5B). Interestingly, the AIP + BMI combination performed noticeably better than the BMI and AIP measurements alone (Figure 5C). For instance, when comparing AIP + BMI to either BMI (NRI: 0.105, 95% CI: 0.042–0.168) or AIP (NRI: 0.173, 95% CI: 0.110–0.236), continuous NRIs were significant.

### Mediation analysis

**Figure 6:**
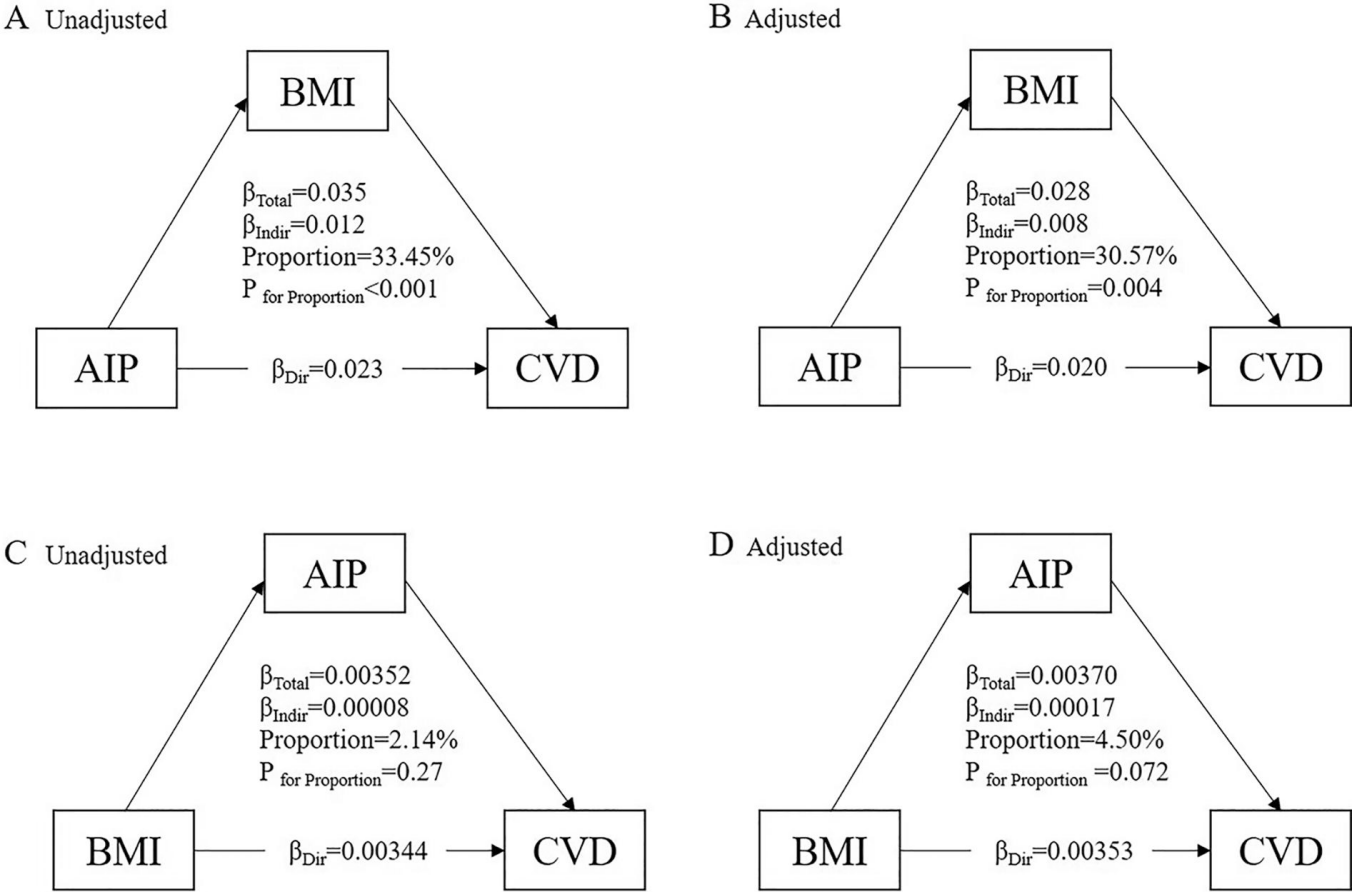
Mediation effect of BMI between the AIP and cardiovascular diseases. **(A)** The unadjusted mediation model for the pathway AIP → BMI → CVD. **(B)** The adjusted mediation model for the pathway AIP → BMI → CVD. **(C)** The unadjusted mediation model for the pathway BMI → AIP → CVD. (D) The adjusted mediation model for the pathway BMI → AIP → CVD.

Figure 6 illustrates the potential mediating effect of increased BMI on the relationship between AIP and cardiovascular diseases. The mediation proportions attributed to increased BMI were 33.45% (*P* < 0.001) in the unadjusted analysis and 30.57% (*P* = 0.004) in the adjusted analysis. However, we did not identify a significant mediating effect of the AIP on the association between increased BMI and cardiovascular diseases. To further verify the robustness of the mediation analysis results, we performed a stratified analysis based on the population's glucose metabolism status. Supplementary Figures S2 and S3 show that regardless of the population's glucose metabolism status, the relationship between AIP and cardiovascular disease is mediated by elevated BMI.

## Discussion

Higher baseline AIP and BMI were considerably positively connected with an elevated risk of cardiovascular diseases, per a 9.0-year follow-up study involving 5,468 Chinese adults over 45. Furthermore, participants with elevated AIP levels also had a notably higher BMI. Those individuals with both elevated AIP and a BMI exceeding 24 kg/m^2^ demonstrated the highest risk for cardiovascular diseases. Even after applying the IPTW approach and controlling for other known risk factors for cardiovascular diseases, this connection persisted in being significant. The combined application of the AIP and BMI was significantly more effective than considering either indicator individually in predicting cardiovascular disease outcomes. Additionally, our study demonstrated that BMI played a major mediating role in the relationship between AIP and cardiovascular diseases.

Low-density lipoprotein cholesterol (LDL-C) levels are crucial in preventing atherosclerosis, which is caused by lipid metabolism abnormalities. However, even when LDL-C levels are reduced, the risk of cardiovascular illnesses persists (14). sdLDL-C is more likely to cause atherosclerosis, yet methods to measure its diameter are complex and costly (15). The value of the AIP is negatively correlated with the particle diameter of sdLDL-C, which may reflect the degree of atherosclerosis (3, 16).

An elevated risk of cardiovascular diseases is substantially correlated with a higher baseline AIP. AIP serves as a reflection of lipid metabolism status, with elevated levels indicative of abnormal lipoprotein profiles. As a result, the incidence and development of cardiovascular diseases are strongly correlated with the AIP. In developing nations, the AIP may be used as an early biomarker for cardiovascular disease, according to a cross-sectional study that included 340 healthy Mexican women (17). A long-term follow-up study of 14,063 Americans revealed that high AIP was related to diabetes mortality in women over 65 years of age and all-cause mortality (18). According to cross-sectional research of 1,000 Iranians, the AIP could forecast the probability of coronary cardiovascular disease and atherosclerosis (19). Research on morbidly obese individuals has suggested that the AIP could predict cardiovascular disease risk, which decreases after surgery (20). The AIP was linked to the general population's risk of ischemic stroke, according to the Kailuan study (21). Multiple epidemiological studies have extensively investigated the association between AIP and established cardiovascular risk factors. Elevated levels of AIP are strongly associated with obstructive sleep apnea, insulin resistance, type 2 diabetes, newly diagnosed hypertension, carotid atherosclerosis, and obesity (6, 22–27).

Most existing studies on AIP and cardiovascular diseases have focused solely on establishing direct associations, leaving a critical gap in understanding how AIP contributes to cardiovascular diseases risk. Our study addresses this gap by delineating and quantifying the mediating role of BMI, supported by both pathophysiological rationale and rigorous statistical validation. From a pathophysiological perspective, the associations between AIP and cardiovascular diseases are complex. The AIP measures the ratio of TG to HDL-C; greater values signify a higher risk of cardiovascular disease (19). Elevated TG promotes vascular endothelial dysfunction and atherosclerosis, whereas reduced HDL-C weakens protection against these conditions (28, 29). BMI plays a mediating role by increasing adipose tissue, which secretes inflammatory factors that disrupt lipid metabolism and vascular function (30–32). This mechanism is further supported by prior evidence linking AIP to obesity-related biomarkers: serum adipocyte-fatty acid binding protein (FABP) and asymmetric dimethylarginine (ADMA) are strongly positively correlated with the AIP (17). Elevated ADMA levels may contribute to obesity via multiple mechanisms, such as interfering with insulin signal transduction and glucose metabolism, activating CaSR to promote lipid accumulation, and disrupting hepatic lipid metabolism (33–35). FABP4 can also facilitate obesity by enhancing fatty acid uptake and transport, regulating adipocyte differentiation, and being involved in inflammatory responses and insulin resistance, resulting in adipocyte hypertrophy (36–38). These findings explain why participants with elevated AIP in our study also had significantly higher BMI, and why BMI emerged as a key mediator. To validate this mediation effect, we employed rigorous statistical methods: 1,000 iterations of bootstrap resampling confirmed the significance of the indirect effect of AIP on cardiovascular diseases via BMI. Additionally, IPTW adjustment minimized selection bias, ensuring the mediation effect was not driven by baseline differences between groups. Together, these results provide the first quantitative evidence that BMI mediates a substantial portion of the AIP- cardiovascular diseases association in Chinese middle-aged and older adults.

Our core findings regarding the associations of the AIP, BMI, and cardiovascular diseases risk align with those of recent pivotal studies, while also addressing critical gaps in mechanistic understanding and study scope—thereby advancing the framework of AIP-related cardiovascular diseases risk research. Compared with the study by Zhao et al. (39), our study corroborates this association using a longer follow-up period and a comparable cohort size to observe a robust positive relationship between baseline AIP and incident cardiovascular diseases, which remained significant even after applying IPTW and adjusting for conventional cardiovascular diseases risk factors (*P* < 0.001). This cross-study consistency reinforces AIP's validity as a reliable cardiovascular diseases risk marker in Chinese middle-aged and older populations. Notably, our study differs from Zhao et al (39). They only explored the direct association between AIP and cardiovascular diseases, without investigating underlying mediating pathways. In contrast, ours is the first study to quantify the mediating role of BMI in the AIP- cardiovascular diseases relationship within a large national cohort of middle-aged and older Chinese adults. We found that approximately 30.57% of the AIP-related cardiovascular diseases risk was mediated by elevated BMI. Min et al (40). found highlight AIP highlights its relevance in metabolically high-risk subgroups, which aligns with our observation that AIP correlates with markers of metabolic dysfunction. However, our study expands on this work by including a broader, community-based population, which is not limited to those with abnormal glucose metabolism, and conducting stratified analyses to explore whether glucose metabolism status modifies the BMI-mediated AIP-CVD pathway. As shown in our Supplementary Figures S2, S3, the mediating effect of BMI was consistent across individuals with normal glucose regulation (mediation proportion: 34.38%) and abnormal glucose metabolism (34.96%). This indicates that glucose metabolism status does not alter the mechanistic link between AIP, BMI, and cardiovascular diseases, extending the clinical applicability of combined AIP-BMI assessment beyond metabolically impaired subgroups.

Our findings have direct practical value for cardiovascular diseases risk stratification and targeted prevention, particularly in primary care and resource-limited settings. First, the combined assessment of AIP and BMI significantly improves cardiovascular diseases risk stratification. Individuals with both elevated AIP and BMI had a higher cardiovascular diseases risk than those with normal AIP and BMI (1.789, 95%CI: 1.491–2.147), accounting for 26.57% of our cohort. This high-risk subgroup represents a priority for intervention, as they may benefit from dual-targeted strategies: weight management and lipid-lowering interventions. Second, AIP serves as a cost-effective surrogate for sdLDL-C. While sdLDL-C is more atherogenic than conventional LDL-C, its measurement requires complex and expensive techniques (15). In contrast, AIP is calculated using routine TG and HDL-C measurements and is negatively correlated with sdLDL-C particle diameter (3, 16), making it accessible for large-scale screening in settings where advanced lipid testing is unavailable. Third, the consistency of the BMI-mediated pathway across glucose metabolism subgroups (Supplementary Figures S2, S3) means combined AIP-BMI assessment can be applied broadly, not just to individuals with abnormal glucose metabolism. This expands its utility beyond Min et al.'s (40) focus, allowing clinicians to identify high-risk individuals across diverse metabolic phenotypes. Notably, the ROC analysis in Figure 5 is an exploratory supplementary analysis focused on the core variables of the mediation model (AIP and BMI), not a dedicated predictive model. Its AUC (0.572) reflects the limitations of using only two mechanistic variables for prediction, rather than negating the value of the AIP-BMI mediation pathway. Future predictive models should integrate this mediation mechanism with other critical risk factors to improve accuracy.

However, the study has certain limitations. First, as an observational study, we cannot establish definitive causality between AIP, BMI, and cardiovascular diseases. While we used IPTW and bootstrap resampling to minimize bias, residual confounding from unmeasured variables may persist. Second, the application of exclusion criteria resulted in a limited sample size and potential attrition bias, though the cohort remained representative of Chinese middle-aged and older adults. Third, cardiovascular diseases diagnoses were based on self-reports, which may introduce misclassification bias. Fourth, our focus on Chinese middle-aged and older adults limits the generalizability of findings to younger populations or other ethnic groups. Fifth, we did not investigate longitudinal variations in AIP, which may underestimate the dynamic interplay between AIP changes and BMI over time.

To address these gaps, future research should: (1) conduct prospective interventional trials to test whether reducing BMI mitigates AIP-related cardiovascular diseases risk, validating the causal relevance of our mediation findings; (2) explore longitudinal AIP-BMI interactions to determine how sustained AIP elevation and BMI changes jointly influence cardiovascular diseases risk; (3) validate the AIP-BMI mediation pathway in diverse cohorts; and (4) integrate the AIP-BMI mediation mechanism into comprehensive predictive models with additional risk factors to enhance clinical utility.

## Conclusions

In conclusion, our study confirms a positive association between AIP and cardiovascular diseases, quantifies the mediating role of BMI in this pathway, and demonstrates the clinical value of combined AIP-BMI assessment. we address key mechanistic gaps and provide evidence for more precise cardiovascular diseases risk stratification and prevention strategies.

## Data Availability

The original contributions presented in the study are included in the article/Supplementary Material, further inquiries can be directed to the corresponding authors.
