# Supplementary material for: Association between the atherogenic index of plasma, body mass index, and cardiovascular diseases in Chinese middle and old-aged adults: a mediation analysis
Source: Front Cardiovasc Med. 2025 Oct 29;12:1597749. doi: 10.3389/fcvm.2025.1597749 (PMC12606225; doi:10.3389/fcvm.2025.1597749)
Supplement: Supplementary file 1 [file Datasheet1.docx]

**Supplemental methods**

**Supplement Study population**

The China Health and Retirement Longitudinal Study (CHARLS) primarily encompasses home visits and physical examinations, with data collected from 28 provinces and 150 counties nationwide. A range of information was gathered, including health details, demographic statistics, socioeconomic status, and physical and physiological measurements. Additional information regarding CHARLS can be accessed on the official website (https://charls.pku.edu.cn/) or through its published works(1).

The baseline survey for CHARLS was conducted between 2011 and 2012 employing a multistage probability sampling methodology (wave 1). To date, CHARLS has published four waves of follow-up data: wave 2 in 2013, wave 3 in 2015, wave 4 in 2018, and wave 5 in 2020.

**Supplement exposure and outcome**

Following a 15-minute rest period, participants were instructed to measure their blood pressure on the left arm three times at 45-second intervals and report the average of these readings; this procedure was exempt for those with arm injuries. The participants were required to remove their shoes and heavy clothing before being weighed and their height measured. Weight and height measurements were obtained using a calibrated scale with accuracies of 0.1 kg and 0.1 cm, respectively. Venous blood samples were collected from each participant by medically trained personnel by established phlebotomy protocols, followed by biochemical analyses. Triglycerides (TG) and high-density lipoprotein cholesterol (HDL-C) levels were determined utilizing enzymatic colorimetric assays, where the coefficient of variation for TG within the assay was 1.5%, whereas that for HDL-C was calculated at 1.0%. Glycosylated hemoglobin (HbA1c) was assessed using boronate affinity high-performance liquid chromatography (HPLC), which revealed a coefficient of variation of 1.9% within the assay framework. Serum creatinine concentrations were measured via the rate-reduced compensated Jaffe method, which demonstrated a coefficient of variation below 1.6%. Uric acid levels were evaluated through the UA Plus method, yielding a within-assay coefficient of variation for uric acid of 1.10%. High-sensitivity C-reactive protein (hs-CRP) was quantified using an immunoturbidimetric assay, which revealed an acceptable coefficient of variation of<1.3%. The overall variability in blood marker measurements remained below 5%.

Cardiovascular diagnosis information was collected using a standardized question: “Have you been told by a doctor that you have been diagnosed with a heart attack, coronary heart disease, or stroke?” The results were assessed by rigorously trained interviewers using standardized questionnaires harmonized with the leading international surveys on aging in the Health and Retirement Study (HRS) and related international surveys on aging, including the English Longitudinal Study of Aging (ELSA) and the Survey of Health, Aging, and Retirement in Europe (SHARE). Quality control of the data collection and verification was performed to ensure the reliability of the data.

**Supplement covariates**

Sex was divided into "male" and "female". Education level was categorized as "primary education or lower" and "high school or higher". Smoking status was defined as "never smoking" or "smoking". The diagnosis of hypertension was based on self-reported physician diagnosis and/or a mean systolic/diastolic blood pressure (SBP/DBP) ≥140/90 mmHg(2). Diabetes was defined as fasting plasma glucose (FPG) ≥126mg/dl or HbA1c ≥6.5% and/or a self-reported physician diagnosis. Pre-diabetes was characterized by an FPG of 100 to 125 mg/dL or an HbA1c of 5.7–6.4%. Normal glucose regulation was defined as FPG<100 mg/ dL and HbA1c<5.7%(3). Diabetes and Pre-Diabetes are collectively referred to as individuals with abnormal glucose metabolism. The eGFR value was calculated according to the 2021 Chronic Kidney Disease Epidemiology Collaboration (CKD-EPI). eGFR (mL/min.1.73m^2^) = 142× (Scr/A)^B^×(0.9938)^Age^ ×C (Note: A refers to the following values by sex: female =0.7, male =0.9. C refers to the following values by sex: female =1.012, male =1. B is defined as the following values by sex and serum creatinine (Scr): female: Scr≤0.7 mg/dl, B=-0.241, Scr>0.7 mg/dl, B=-1.2; male: Scr≤ 0.9 mg/dl, B=-0.302, Scr>0.9 mg/dl =-1.2. Scr was calculated as 1 mg/dl=88.49 µmol/L(4).

**Supplemental statistical analyses**

The AIP group (defined as having a value below the median of 0.72) served as the predictor variable (X), whereas increased BMI (≥24 kg/m²) was designated the mediator variable (M). Cardiovascular disease onset represented the outcome variable (Y). The analysis comprises four key steps: (1) establish that X is associated with Y (Model Y = βₜₒₜ X), where βₜₒₜ denotes the total effect; (2) demonstrate that X is correlated with M (Model M = β₁ X), where β₁ represents an indirect effect 1; (3) determine which portion of Y can be explained by controlling for X in Model Y = β₂ M + βᴅɪʀ X, with β₂ an indicating indirect effect and βᴅɪʀ denoting a direct effect; and finally, (4) calculate the proportion of the indirect or mediation effect using the following: mediation effect (%) = (β₁ × β₂ / βₜₒₗ) × 100%. This methodological approach has been frequently employed in previous studies to quantify mediating effects(5).

**Supplement figures**


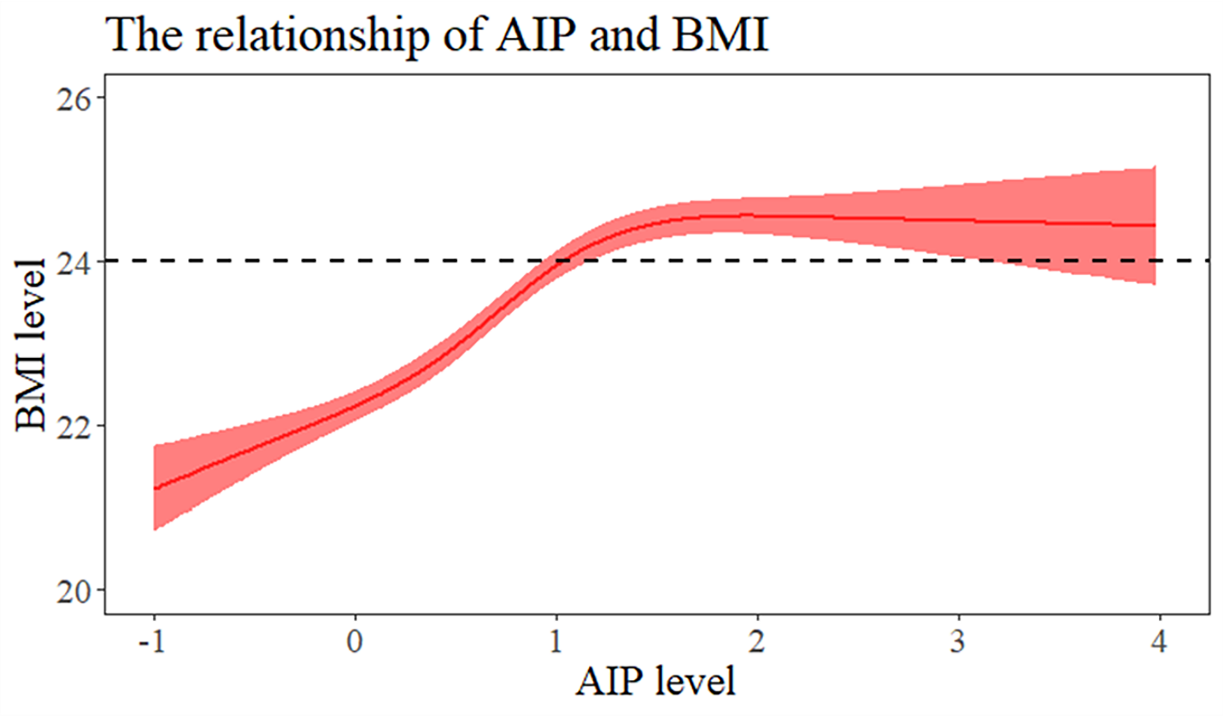


**Figure. S1** Dose-responsive relationship of the AIP and BMI levels.


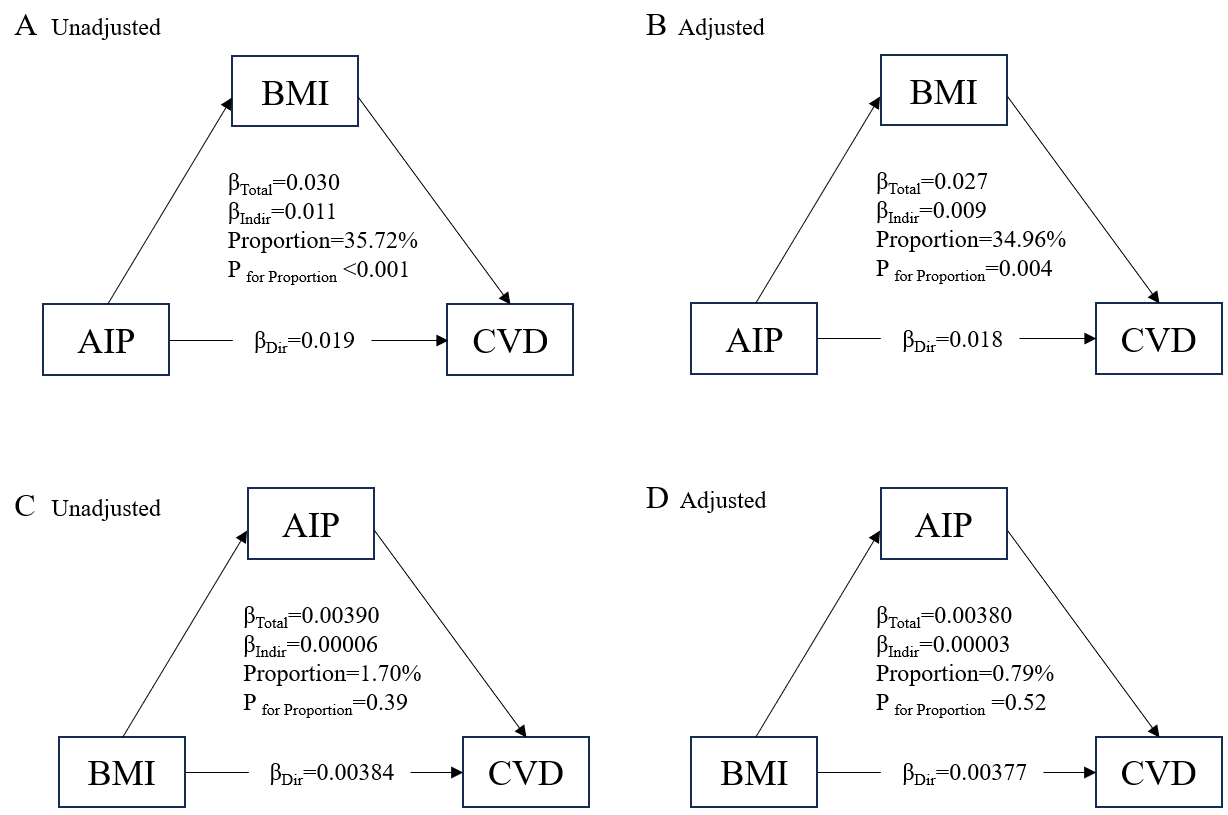
**Figure. S2** Mediation effect of BMI between the AIP and cardiovascular diseases for individuals with abnormal glucose metabolism.


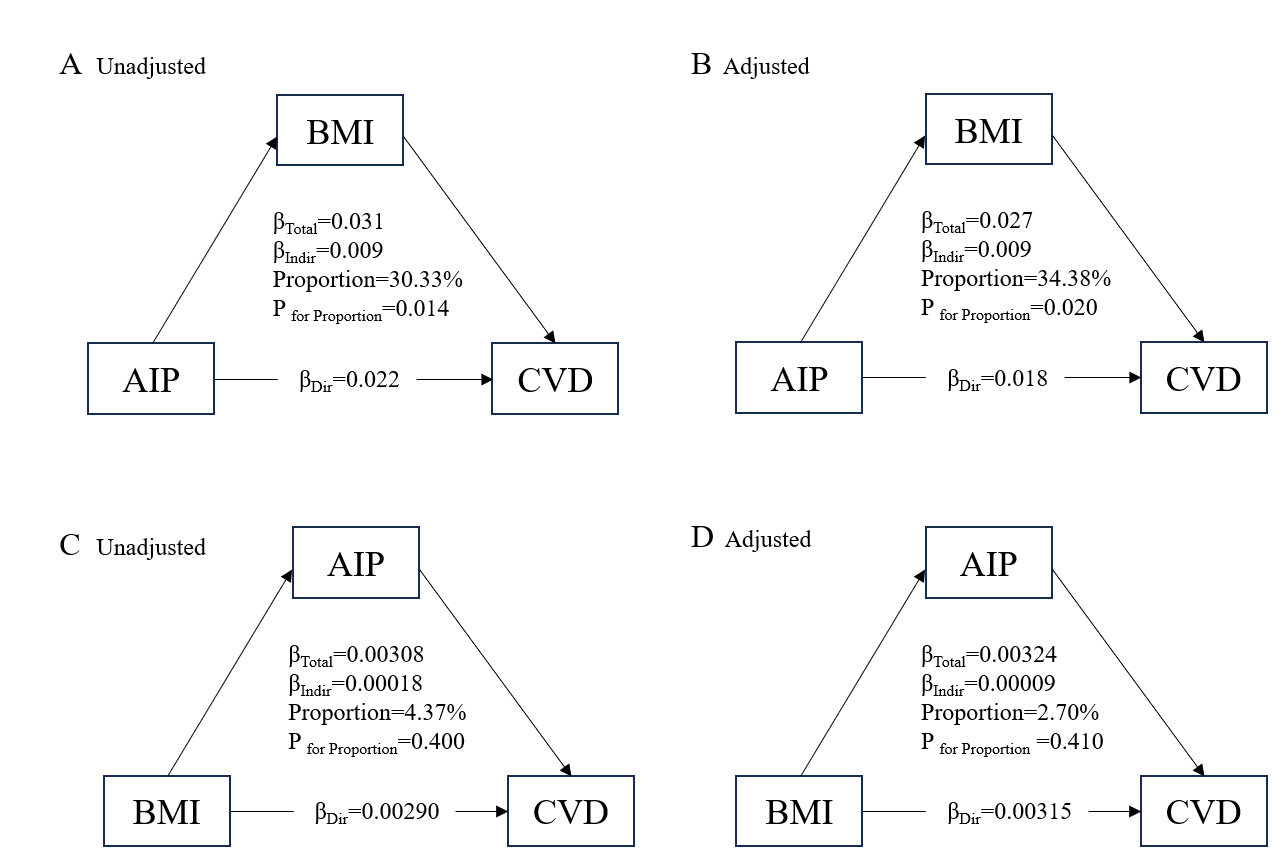


**Figure. S3** Mediation effect of BMI between the AIP and cardiovascular diseases for individuals with normal glucose regulation

**Supplement tables**

| **Table S1**: Characteristics of 5468 participants categorized by cardiovascular diseases | | | | |  |
| --- | --- | --- | --- | --- | --- |
|  | Overall | CVD=0 | CVD=1 | P-Value | |
| Participants, No | 5468 | 4220 | 1248 |  | |
| Age, years, mean (SD) | 58.2(8.6) | 57.5(8.5) | 60.3(8.8) | <0.001 | |
| Sex, Female, n (%) | 2957(54.1) | 2244(53.2) | 713(57.1) | 0.016 | |
| Residence, n (%) |  |  |  | <0.001 | |
| Rural | 4728(86.5) | 3675(87.1) | 1053(84.4) | 0.018 | |
| Urban | 738(13.5) | 544(12.9) | 194(15.6) |  | |
| Marriage, married, n (%) | 4940(90.3) | 3847(91.2) | 1093(87.6) | <0.001 | |
| Educational level, n (%) |  |  |  | 0.002 | |
| High school or higher | 524(9.6) | 397(9.4) | 127(10.2) |  | |
| Primary or lower | 4943(90.4) | 3822(90.6) | 1121(89.8) |  | |
| Smoking status, n (%) |  |  |  | 1.000 | |
| Never | 3391(62.1) | 2617(62.1) | 774(62.1) |  | |
| Smoking | 2073(37.9) | 1600(37.9) | 473(37.9) |  | |
| Drinking status, n (%) |  |  |  | 0 .500 | |
| Never | 3751(92.6) | 2872(92.8) | 879(92.0) |  | |
| Drinking | 300(7.4) | 224(7.2) | 76(8.0) |  | |
| Hypertension, n (%) | 1921(35.3) | 1334(31.8) | 587(47.3) | <0.001 | |
| Diabetes, n (%) | 839(15.5) | 594(14.2) | 245(19.8) | <0.001 | |
| Height, cm, mean (SD) | 157.9(8.5) | 157.9(8.4) | 157.8(8.6) | 0.711 | |
| Weight, kg, mean (SD) | 58.6(11.0) | 58.1(10.7) | 60.3(11.8) | <0.001 | |
| Waist, cm, mean (SD) | 84.9(9.8) | 84.2(9.5) | 87.2(10.4) | <0.001 | |
| SBP, mmHg, mean (SD) | 128.7(20.5) | 127.2(19.9) | 133.7(22.0) | <0.001 | |
| DBP, mmHg, mean (SD) | 75.2(11.9) | 74.5(11.6) | 77.3(12.6) | <0.001 | |
| Glucose, mg/dL, mean (SD) | 109.2(34.3) | 107.7(30.2) | 114.1(45.0) | <0.001 | |
| HbA1c, %，mean(SD) | 5.2(0.8) | 5.2(0.7) | 5.4(1.0) | <0.001 | |
| C-Reactive Protein, mg/dL, mean (SD) | 2.4(6.7) | 2.3(6.7) | 2.8(6.6) | 0.033 | |
| Creatinine, mg/dL, mean (SD) | 0.8(0.2) | 0.8(0.2) | 0.8(0.2) | 0.163 | |
| Uric acid, mg/dL, mean (SD) | 4.4(1.2) | 4.4(1.2) | 4.4(1.3) | 0.217 | |
| eGFR, mL/min/1.73m^2^, mean (SD) | 97.0(13.4) | 97.6(13.2) | 94.9(13.9) | <0.001 | |
| Triglycerides, mg/dL, mean (SD) | 132.5(112.9) | 129.8(110.9) | 141.6(119.1) | 0.001 | |
| Total Cholesterol, mg/dL, mean (SD) | 193.8(38.7) | 192.9(38.7) | 196.7(38.5) | 0.002 | |
| HDL Cholesterol, mg/dL, mean (SD) | 51.5(15.2) | 52.0(15.2) | 50.1(14.9) | <0.001 | |
| LDL Cholesterol, mg/dL, mean (SD) | 116.4(34.7) | 115.6(34.4) | 118.9(35.5) | 0.004 | |
| AIP, mean (SD) | 0.8(0.8) | 0.8(0.8) | 0.9(0.8) | <0.001 | |
| BMI, kg/m^2^, mean (SD) | 23.4(3.6) | 23.2(3.5) | 24.1(3.9) | <0.001 | |

Data are presented as mean (SD) or number (%), as appropriate.

Abbreviation: SD, standard deviation; AIP, atherogenic index of plasma; BMI, body mass index; SBP, systolic blood pressure; DBP, diastolic blood pressure; HbA1c, glycosylated hemoglobin; eGFR, estimated glomerular filtration rate; LDL, low-density lipoprotein; HDL, high-density lipoprotein; CVD, cardiovascular diseases. CVD = 1 indicates that the primary endpoint events occurred during the follow-up period, and CVD = 0 indicates that the endpoint event did not occur during the follow-up period.

| **Table S2:** Associations of AIP, BMI levels with risk of cardiovascular diseases | | | | |
| --- | --- | --- | --- | --- |
|  | Unadjusted | | Adjusted | |
|  | HR (95% CI) | P value | HR (95% CI) | P value |
| AIP< median | Ref |  | Ref |  |
| AIP ≥ median | 1.464(1.282-1.670) | <0.001 | 1.359(1.184-1.562) | <0.001 |
| BMI <24 | Ref |  | Ref |  |
| BMI ≥24 | 1.507(1.323-1.717) | <0.001 | 1.483(1.287-1.708) | <0.001 |

BMI was calculated by dividing an individual's weight in kilograms by the square of their height in meters; the unit of BMI: kg/m^2^; the median value of the AIP: 0.72. Age, gender, education level, smoking status, hypertension, diabetes, HbA1c, uric acid, and eGFR were adjusted. HR: hazard ratio, CI: confidence interval, BMI: body mass index, AIP: atherogenic index of plasma.

|  | Sensitivity analyses 1 | | Sensitivity analyses 2 | | | Sensitivity analyses 3 | |  |
| --- | --- | --- | --- | --- | --- | --- | --- | --- |
|  | HR (95% CI) | P value | HR (95% CI) | P value | HR (95% CI) | | P value | |
| AIP< median & BMI <24 | Ref |  |  |  |  | |  | |
| AIP< median & BMI ≥24 | 1.651(1.326-2.056) | <0.001 | 1.629(1.308-2.029) | <0.001 | 1.627(1.267-2.089) | | <0.001 | |
| AIP ≥ median & BMI <24 | 1.418(1.179-1.704) | <0.001 | 1.401(1.165-1.685) | <0.001 | 1.444(1.178-1.771) | | <0.001 | |
| AIP ≥ median & BMI ≥24 | 1.789(1.491-2.147) | <0.001 | 1.756(1.464-2.106) | <0.001 | 2.830(1.490-2.248) | | <0.001 | |

**Table S3:** Sensitivity analyses of the combination assessment of the AIP and BMI levels with the risk of cardiovascular diseases

Age, gender, education level, smoking status, hypertension, diabetes, HbA1c, eGFR, and uric acid were adjusted. Sensitivity analyses 1: additionally adjusted for hs-CRP level; sensitivity analyses 2: using 130/80 mmHg to define hypertension; sensitivity analyses 3: using propensity scores of inverse probability treatment weighting (IPTW) method.

| **Table S4** Associations of AIP and BMI levels with risk of heart disease | | | | |
| --- | --- | --- | --- | --- |
|  | Unadjusted | | Adjusted | |
|  | HR (95% CI) | P value | HR (95% CI) | P value |
| AIP< median & BMI <24 | Ref |  | Ref |  |
| AIP< median & BMI ≥24 | 1.730(1.367-2.189) | <0.001 | 1.747(1.370-2.228) | <0.001 |
| AIP ≥ median & BMI <24 | 1.480(1.205-1.818) | <0.001 | 1.410(1.143-1.739) | 0.001 |
| AIP ≥ median & BMI ≥24 | 1.846(1.526-2.233) | <0.001 | 1.771(1.440-2.177) | <0.001 |

BMI was calculated by dividing an individual's weight in kilograms by the square of their height in meters; the unit of BMI: kg/m2; the median value of the AIP: 0.72. Age, sex, education level, smoking status, hypertension, diabetes, HbA1c, uric acid, and eGFR were adjusted. HR: hazard ratio, CI: confidence interval, BMI: body mass index, AIP: atherogenic index of plasma.

| **Table S5:** Associations of AIP and BMI levels with risk of stroke | | | | |
| --- | --- | --- | --- | --- |
|  | Unadjusted | | Adjusted | |
|  | HR (95% CI) | P value | HR (95% CI) | P value |
| AIP< median & BMI <24 | Ref |  | Ref |  |
| AIP< median & BMI ≥24 | 1.365(0.882-2.112) | 0.163 | 1.439(0.914-2.265) | 0.116 |
| AIP ≥ median & BMI <24 | 1.520(1.072-2.157) | 0.019 | 1.468(1.029-2.094) | 0.034 |
| AIP ≥ median & BMI ≥24 | 1.778(1.277-2.475) | <0.001 | 1.652(1.151-2.372) | 0.006 |

BMI was calculated by dividing an individual's weight in kilograms by the square of their height in meters; the unit of BMI: kg/m^2^; the median value of the AIP: 0.72. Age, sex, education level, smoking status, hypertension, diabetes, HbA1c, uric acid, and eGFR were adjusted. HR: hazard ratio, CI: confidence interval, BMI: body mass index, AIP: atherogenic index of plasma.

**Table S6:** Sensitivity analysis of the combination assessment of the AIP and BMI levels on cardiovascular diseases risk under different BMI cut-off values

|  | Sensitivity analyses 1 | | Sensitivity analyses 2 | |
| --- | --- | --- | --- | --- |
|  | HR (95% CI) | P value | HR (95% CI) | P value |
| AIP< median & BMI < cut-off value | Ref |  | Ref |  |
| AIP< median & BMI ≥ cut-off value | 1.606(1.261-2.045) | <0.001 | 1.357(1.101-1.673) | 0.004 |
| AIP ≥ median & BMI < cut-off value | 1.311(1.105-1.555) | 0.002 | 1.436(1.173-1.757) | <0.001 |
| AIP ≥ median & BMI ≥ cut-off value | 1.881(1.565-2.261) | <0.001 | 1.623(1.351-1.951) | <0.001 |

Age, gender, education level, smoking status, hypertension, diabetes, HbA1c, eGFR, and uric acid were adjusted. Sensitivity analyses 1: using BMI ≥25kg/m^2^; to define overweight and obesity. Sensitivity analyses 2: using BMI ≥23kg/m^2^; to define overweight and obesity

**Supplement** **references**

1. Zhao Y, Hu Y, Smith JP, Strauss J, Yang G. Cohort profile: the China Health and Retirement Longitudinal Study (CHARLS). Int J Epidemiol. 2014;43(1):61-8.

2. Lenfant C, Chobanian AV, Jones DW, Roccella EJ. Seventh report of the Joint National Committee on the Prevention, Detection, Evaluation, and Treatment of High Blood Pressure (JNC 7): resetting the hypertension sails. Hypertension. 2003;41(6):1178-9.

3. ElSayed NA, Aleppo G, Aroda VR, Bannuru RR, Brown FM, Bruemmer D, et al. 2. Classification and Diagnosis of Diabetes: Standards of Care in Diabetes-2023. Diabetes Care. 2023;46(Suppl 1):S19-s40.

4. Inker LA, Eneanya ND, Coresh J, Tighiouart H, Wang D, Sang Y, et al. New Creatinine- and Cystatin C-Based Equations to Estimate GFR without Race. N Engl J Med. 2021;385(19):1737-49.

5. Wu Z, Jiang Y, Zhou D, Chen S, Zhao Y, Zhang H, et al. Sex-specific Association of Subclinical Hypothyroidism With Incident Metabolic Syndrome: A Population-based Cohort Study. J Clin Endocrinol Metab. 2022;107(6):e2365-e72.
